# The potential role of environmentally associated DNA methylation in childhood acute lymphoblastic leukaemia subtypes

**DOI:** 10.1002/ijc.35506

**Published:** 2025-06-09

**Authors:** Jessica R. Saville, Lisa J. Russell, Kay Padget, Akram Ghantous, Jessica Nordlund, Jill A. McKay

**Affiliations:** ^1^ Faculty of Health and Life Sciences, Department of Applied Sciences Northumbria University Newcastle upon Tyne UK; ^2^ Biosciences Institute, Newcastle University Centre for Cancer, Faculty of Medical Science Newcastle University Newcastle upon Tyne UK; ^3^ St. George's University, Grenada, Based at Northumbria University Newcastle upon Tyne UK; ^4^ Epigenomics and Mechanisms Branch International Agency for Research on Cancer Lyon France; ^5^ Department of Medical Sciences, Molecular Precision Medicine, Science For Life Laboratory Uppsala University Uppsala Sweden

**Keywords:** day care attendance, epigenetic, maternal caffeine, maternal folate, maternal radiation exposure, maternal smoking, reported colds

## Abstract

Various genetic aberrations are suggested to initiate the development of acute lymphoblastic leukaemia (ALL) but alone are insufficient for disease onset. Epigenetic alteration, such as DNA methylation changes, plays a key role in human health. Evidence suggests DNA methylation may be an intermediate mechanism through which the environment contributes to ALL manifestation. ALL is categorized into subtypes based on leukaemia‐associated genetic events, and it is plausible that different exposures pose differing risks for given subtypes. Using our previously established meet‐in‐the‐middle approach, we performed CpG‐level analysis to investigate DNA methylation as an intermediate mechanism between risk exposures and ALL. Differentially methylated CpGs (DMCs) were integrated, identifying overlapping methylation, with hypergeometric tests used to assess the probability of concurring methylation considering directionality. DMC analysis reinforced previous gene‐level findings suggesting altered DNA methylation associated with maternal radiation exposure, alcohol intake, and plasma folate during pregnancy is also present in the disease. Whilst maternal folate‐associated and leukaemia‐associated methylation appear consistent across most subtypes, the effect of other exposures appears subtype‐specific. We suggest environmentally associated methylation includes driver and/or ‘navigator’ changes, the latter influencing biological pathways contributing to ALL. This analysis aids understanding of which risk factors may contribute to specific subtypes or which influence ALL risk more generally.

## What's New?

The rarity of acute lymphoblastic leukaemia and lack of available environmental data have made the role of environmental factors in genetic subtypes challenging to investigate. Here, the authors performed a CpG‐level analysis to investigate DNA methylation as an intermediate mechanism between environmental risk exposures and acute lymphoblastic leukaemia. The results suggest that altered DNA methylation associated with maternal radiation exposure, maternal alcohol intake, and maternal plasma folate levels may contribute to disease aetiology. Whilst maternal folate‐associated and leukaemia‐associated methylation were consistent across most subtypes, the effect of other exposures appeared subtype‐specific. Environmentally associated methylation may influence biological pathways contributing to acute lymphoblastic leukaemia.

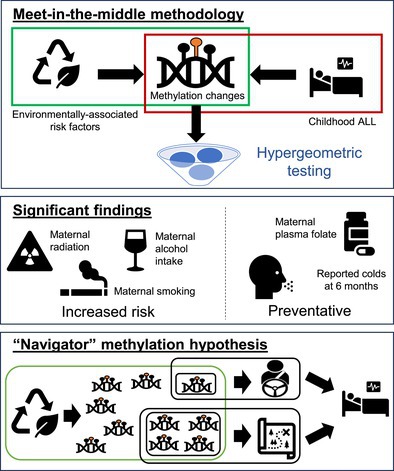

**Abbreviations**
ALL: acute lymphoblastic leukaemia
BCP‐ALL: B‐cell precursor ALL
DMC: differentially methylated CpG
EWAS: epigenome‐wide association studies
HeH: high‐hyperdiploidy
KEGG: Kyoto Encyclopaedia of Genes and Genomes
T‐ALL: T‐cell precursor ALL

## 1 INTRODUCTION

The aetiology of childhood acute lymphoblastic leukaemia (ALL) remains unclear. Whilst genetic aberrations such as chromosomal translocations and aneuploidy have been suggested to be the initiating events in disease development, alone they are not sufficient for disease manifestation. For example, it has been suggested that the *ETV6::RUNX1* gene fusion is present in around 5% of healthy newborns, which is at a 500‐times higher rate than the number of ALL cases associated with that genetic event.^1^ Therefore, additional spontaneous or environmentally induced factors or ‘hits’ are likely to be key for disease development.

Incidence rates of ALL are slowly increasing^2^ suggesting environmental factors contribute to these additional ‘hits’ required for disease manifestation. Indeed, ALL risk has been associated to various extents with a range of exposures experienced *in utero* ^3^ (e.g. smoke exposure,^4^ alcohol,^5^, ^6^ caffeine,^5^, ^7^, ^8^ folic acid,^9^, ^10^, ^11^, ^12^ radiation,^13^, ^14^ household paints,^15^ chemicals,^16^ pesticides,^17^, ^18^ and herbicides^18^, ^19^) and throughout early childhood (e.g. breast feeding,^20^ infection history,^21^ and childcare/day care attendance^20^, ^21^). However, the mechanisms through which these exposures may contribute to risk remain unclear.

Epigenetic alteration, that is changes in DNA methylation, plays a key role in human health and disease through control of gene expression and is one mechanism by which exposures could contribute to disease.^22^ DNA methylation patterns are also dysregulated in ALL itself.^23^ Previously, we provided evidence suggesting DNA methylation may be an intermediate mechanism through which some environmental factors may contribute to ALL manifestation.^3^ We observed directionally concordant gene methylation changes in ALL and in response to maternal radiation exposure, maternal alcohol intake, and maternal sugary caffeinated drink intake during pregnancy, which were not likely to be due to chance. This provided evidence for environmental exposures to drive disease‐associated methylation and could explain one contributing mechanistic pathway towards ALL.

ALL is heterogeneous, arising from B‐cell (BCP‐ALL) or T‐cell precursor haematopoietic lineages (T‐ALL). These immunophenotypes are further defined into subtypes based on the leukaemia‐initiating chromosomal alterations they possess.^24^ BCP‐ALL subtypes include high‐hyperdiploidy (HeH), t(12;21)*ETV6::RUNX1*, t(1;19)*E2A::PBX1*, t(9;22)*BCR::ABL1*, dic(9::20), iAMP21, hypodiploidy (<45 chr), and *KMT2A* (also known as *MLL1*) rearrangements, amongst others.^24^ T‐ALL is more diverse and complex in terms of genetic lesions, and although 50% of cases have chromosomal translocations present, they are not used for risk stratification as their prognostic value is unclear.^25^ T‐ALL subtypes consist of *TLX1*, *LYL1*, *TAL::LMO2*, and *TLX3*.^25^ Due to the differing genetic profiles for each subtype, it is plausible that different exposures may pose differing levels of risk for any given subtype. Indeed, some evidence suggests that tobacco smoking varies the risk of ALL dependent on subtype.^4^ However, the rarity of ALL, coupled with a lack of available environmental data, means studies are often underpowered to detect associations between exposures and ALL even before subtype stratification, making the environmental aetiology of subtypes challenging to investigate. Examining common biomarkers, such as DNA methylation patterns, may help elucidate which exposures may contribute to disease development or progression for specific subtypes.

Here we employed our previously established meet‐in‐the‐middle approach to first perform a DMC‐based analysis to investigate DNA methylation as an intermediate mechanism between potential risk exposures and ALL, and second, investigate this relationship across specific ALL subtypes.

## 2 METHODS

### 2.1 Data sets utilized for analysis

We utilized data from our previously published epigenome‐wide association studies (EWAS)^3^ and from published meta‐analysis^26^, ^27^ to identify differentially methylated CpGs (DMCs) that are associated with ALL environmental risk factors (Table [Link], [Link]). These include maternal alcohol intake, smoking, radiation exposure, folic acid supplementation/maternal plasma folate, coffee consumption, and sugary caffeinated drink consumption during pregnancy on DNA methylation status in offspring cord blood. For all data sets, DNA methylation was measured using the Illumina Infinium® HumanMethylation450k BeadChip assay with statistically significant DMCs based on Bonferroni correction. For DMCs in ALL and across subtypes, we selected data from the most comprehensive study to date measuring DNA methylation in 776 ALL patients (accession number GSE49031), where analysis of cytogenetic subtype was considered.^23^ In this study, we have labelled DMCs that are conserved across all 10 of the ALL subtypes as ‘constitutive’ DMCs, as described in Nordlund et al.^23^

### 2.2 Integration of DMCs associated with ALL and risk exposures

Figure S1 gives a schematic representation of the overall data set analysis. The bioinformatics and research computing tool (http://barc.wi.mit.edu/tools/compare/) was used to integrate DMCs associated with each ALL subtype and constitutive DMCs with those DMCs with altered methylation associated with risk exposures. This identified DMCs where altered methylation was common either constitutively or in an ALL subtype, and in response to a risk exposure. Where common DMCs were observed, hypergeometric tests were performed within R to identify if observed common methylation changes were likely to be due to chance, using a significance of *p* ≤ 0.05, with *n* = 435,941 (i.e. the total number of DMCs measured in Nordlund et al.^23^) as the population size. Although DNA methylation is binary at an individual locus in an individual cell, when measured, methylation is assessed continually as a percentage, or beta value. Methylation can therefore increase or decrease in response to an exposure or in relation to a control. Where exposures are considered risk factors for ALL (i.e. maternal smoking,^28^ alcohol intake,^5^ radiation exposure,^13^, ^14^ coffee consumption,^29^ sugary caffeinated drink consumption^8^), if DNA methylation directly affected the causal disease pathway, the directionality of methylation change in response to that exposure would be expected to be the same as that observed in ALL. Conversely, the directionality of methylation change for protective exposures (i.e. maternal folate,^9^, ^12^ nursery attendance at 8 months^20^, ^21^ and reported cold symptoms within the first 6 months^21^) would be expected to be opposing to that observed in ALL. Therefore, the directionality of DNA methylation was considered in additional hypergeometric tests contingent on the exposure being a ‘risk’ or ‘protective’ factor, whereby the ‘concordance’ refers to the expected directionality.

### 2.3 Pathway analysis

DAVID (version 2021 released December 2021, accessed between June and August 2022)^30^ was used to assess Kyoto Encyclopaedia of Genes and Genomes (KEGG) pathways affected by subtype‐specific differential DNA methylation and environmentally associated differential DNA methylation. Official gene identifiers for genes mapping to DMCs were used for analysis. The threshold for significance was *p* < 0.05 (uncorrected).

## 3 RESULTS

### 3.1 Integration of constitutive ALL and risk exposure DMCs

In previously published work we used gene‐level methylation, whereby differentially methylated CpGs that are annotated to specific genes associated with the environment or ALL were integrated to identify gene overlaps. In this study we have integrated DMCs at specific CpG sites associated with the environment or ALL to identify DMC overlaps. Hypergeometric tests assessed the probability of relationships between exposure‐associated and ALL‐associated DMCs. Whilst *p*‐values were more modest using the DMC rather than gene‐specific approach, the % concordance between the expected direction of methylation change was higher (in most instances 100%), for all exposures where overlapping DMCs were observed (Table 1). Supporting our previous approach, this more sensitive DMC analysis reinforced our earlier findings indicative of significant overlap between radiation (*p* = 0.05) and alcohol exposure‐related (*p* = 0.025) methylation and changes observed in ALL (Table 1), and lack of association for maternal folate supplementation and coffee consumption during pregnancy. Whilst previously, sugary caffeinated drink intake during pregnancy was observed to have significant overlapping gene methylation with ALL‐associated methylation, here overlaps between DMCs were short of statistical significance (*p* = 0.055), but with increased concordance for directionality of methylation change (100% for DMCs compared with 83% for gene methylation). Previous findings at the gene level suggested smoking‐related methylation significantly overlapped with ALL methylation, but not for concordance of directionality (Table 1). Here, an assessment of DMCs found neither overall nor directionally concordant methylation to be significant. Moreover, whilst previously using data from a meta‐analysis of smoking exposure during pregnancy and offspring methylation in our gene‐level approach, we observed a significant overlap of genes with altered methylation in response to maternal smoking and with altered methylation in ALL. Our DMC analysis revealed that the overlapping methylation observed was likely to be due to chance (Table 2, *p* = 1). Conversely, using data from a meta‐analysis for maternal plasma folate, our DMC analysis corroborated previous findings of our gene‐level approach, not only suggesting that at both the gene and DMC levels that exposure and ALL‐associated methylation are unlikely to be due to chance, but also that at the DMC changes were in 100% concordance for directionality (Table 2).

**TABLE 1:** Number of genes and DMCs with altered methylation due to an ALL‐risk exposure (as identified in Timms et al.) and the overlapping number of genes and DMCs also observed to have altered methylation in ALL across all subtypes.

Exposure	Number (*n*) of DMCs (genes) related to exposure	Overlapping genes between exposure and ALL associated methylation^a^ (*n* [*p*‐value])	Overlapping genes with expected^b^ direction of methylation change (*n* [% concordance; *p*‐value])	Overlapping DMCs between exposure and ALL associated methylation^a^ (*n* [*p*‐value])	Overlapping DMCs with expected^b^ direction of methylation change (*n* [% concordance; *p*‐value])
Maternal radiation during pregnancy	288 (239)	54 (**1.69 × 10** ^**−8**^)	40 (74%; **0.001**)	13 (**0.011**)	11 (85%; **0.050**)
Maternal intake of alcohol intake during pregnancy	192 (175)	33 (**3.98 × 10** ^**−4**^)	29 (88%; **0.006**)	9 (**0.025**)	9 (100%; **0.025**)
Maternal sugary caffeinated drinks intake during pregnancy	66 (54)	12 (**7.42 × 10** ^**−3**^)	10 (83%; **0.045**)	4 (0.055)	4 (100%; 0.055)
Maternal smoking throughout all pregnancy	22 (13)	5 (**7.11 × 10** ^**−3**^)	3 (60%; 0.140)	1 (0.381)	1 (100%; 0.381)
Folic acid supplementation during pregnancy	9 (7)	2 (0.155)	1 (50%; 0.155)	0	0
Maternal coffee intake during pregnancy	15 (13)	1 (0.754)	0	0	0
Reported a cold (6 months)^b^	75 (60)	9 (0.156)	6 (67%; 0.587)	5 (**0.023**)	5^b^ (100%; **0.023**)
Day nursery 8 months	11 (11)	6 (**2.65 × 10** ^**−5**^)	5 (83%; **0.003**)	0	0

*Note*: All *p*‐values are for hypergeometric tests. *P*‐values in bold have <0.05 significance. Columns in grey relating to gene‐level analysis are pre‐published data^3^ shown here for comparison.

^a^ Constitutive methylation across ALL subtypes as reported in Nordlund et al.^23^ 9406 DMCs.

^b^ Where exposures are hypothesized to increase risk, the same direction of methylation change is expected; however, where exposures are hypothesized to be protective, an opposing direction of methylation change is expected.

**TABLE 2:** Number of genes and DMCs with altered methylation due to an ALL‐risk exposure reported in published 450 K studies (FDR corrected and cell type adjusted) and the overlapping number of genes and DMCs also observed to have altered methylation in ALL across all subtypes.

Exposure	Number (*n*) of DMCs (genes) related to exposure	Overlapping genes with exposure and ALL associated methylation^a^ (*n* [*p‐*value])	Overlapping DMCs with exposure and ALL associated methylation^a^ (*n* [*p*‐value])	Overlapping DMCs with expected^b^ direction of methylation change (*n* [% concordance; *p*‐value])
Maternal smoking (sustained)	6073 (3176)	431 (**4.95 × 10** ^**−11**^)	99 (1)	65 (65.7%; 1)
Maternal plasma folate^b^	443 (229)	62 (**4.32 × 10** ^**−13**^)	18 (**0.009**)	17^b^ (94.4%; **0.018**)

*Note*: All *p*‐values are for hypergeometric tests. *P*‐values in bold have <0.05 significance. Columns in grey relating to gene‐level analysis are pre‐published data^3^ shown here for comparison.

^a^ Constitutive methylation across ALL subtypes as reported in Nordlund et al.^23^ 9406 DMCs.

^b^ Where exposures are hypothesized to increase risk, the same direction of methylation change is expected; however, where exposures are hypothesized to be protective, the opposing direction of methylation change is expected.

The most pronounced difference between the gene and DMC analysis was for post‐natal exposures of reported colds and nursery attendance (Table 1). Where previously overlapping gene methylation had been significant for nursery attendance, no DMCs were found in common, suggesting different gene regions have altered methylation associated with nursery attendance than are altered in ALL. Conversely, for reported colds, overlapping DMCs were found to be significant (Table 1, *p* = 0.023), where gene methylation was not. Moreover, the opposing directions of change at DMCs associated with reported colds and methylation in ALL, which should be considered for this proposed protective exposure, were 100%, compared with 67% for gene methylation.

### 3.2 Integration of ALL subtype‐specific and risk exposure DMCs

As observed across all subtypes, there was no significant overlap between subtype‐specific DMCs and DMC changes associated with maternal sugary caffeinated drinks intake, folic acid supplementation, or coffee intake (Table 3).

**TABLE 3:** Number of DMCs with altered methylation due to an ALL‐risk exposure and the overlapping number of DMCs also observed to have altered methylation in individual ALL subtypes.

ALL subtype (number of associated DMCs)	T‐ALL (58157)		*KMT2A‐r (MLL)* (31403)		*dic(9::20)* (53680)		HEH (42779)		*TCF3::PBX1* (21799)	
Exposure (number of associated DMCs)	Overlapping DMCs	Concordant DMCs (*p*‐value)	Overlapping DMCs	Concordant DMCs (*p*‐value)	Overlapping DMCs	Concordant DMCs (*p*‐value)	Overlapping DMCs	Concordant DMCs (*p*‐value)	Overlapping DMCs	Concordant DMCs (*p*‐value)
Radiation (288)	60 (**2.87 × 10** ^**−4**^)	48 (0.061)	38 (**2.43 × 10** ^**−4**^)	29 (**0.044**)	49 (**0.012**)	39 (0.288)	33 (0.200)	‐	22 (**0.033**)	19 (0.135)
Alcohol (192)	39 (**0.005**)	38 (**0.008**)	15 (0.411)	‐	30 (0.102)	‐	24 (0.131)	‐	12 (0.255)	‐
Sugary caffeinated drinks (66)	13 (0.095)	‐	7 (0.196)	‐	11 (0.184)	‐	9 (0.196)	‐	5 (0.234)	‐
Smoking throughout (22)	4 (0.337)	‐	6 (**0.004**)	5 (**0.018**)	7 (**0.014**)	5 (0.125)	5 (0.058)	‐	3 (0.095)	‐
Folic Acid^a^ (9)	1 (0.724)	‐	1 (0.490)	‐	1 (0.694)	‐	1 (0.605)	‐	0 (−)	‐
Coffee (15)	3 (0.323)	‐	0 (−)	‐	0 (−)	‐	0 (−)	‐	0 (−)	‐
Reported cold 6 months^a^ (75)	14 (0.120)	‐	10 (**0.042**)	10 (**0.042**)	13 (0.127)	‐	10 (0.198)	‐	5 (0.321)	‐
Day nursery 8 months^a^ (11)	4 (**0.048**)	0 (−)	4 (**0.006**)	0 (−)	4 (**0.037**)	0 (−)	3 (0.086)	‐	0 (−)	‐

ALL subtype (number of associated DMCs)	*ETV6::RUNX1* (45589)		*BCR::ABL1* (23871)		iamp21 (44726)		Undefined (39262)		Non‐recurrent (42109)	
Exposure (number of associated DMCs)	Overlapping DMCs	Concordant DMCs (*p*‐value)	Overlapping DMCs	Concordant DMCs (*p*‐value)	Overlapping DMCs	Concordant DMCs (*p*‐value)	Overlapping DMCs	Concordant DMCs (*p*‐value)	Overlapping DMCs	Concordant DMCs (*p*‐value)
Radiation (288)	48 (**8.41 × 10** ^**−4**^)	37 (0.112)	19 (0.234)	‐	31 (0.417)	‐	40 (**0.004**)	33 (0.092)	42 (**0.005**)	32 (0.228)
Alcohol (192)	29 (**0.028**)	28 (**0.045**)	12 (0.361)	‐	22 (0.325)	‐	25 (**0.040**)	24 (0.064)	25 (0.077)	‐
Sugary caffeinated drinks (66)	9 (0.250)	‐	6 (0.152)	‐	10 (0.135)	‐	8 (0.241)	‐	8 (0.304)	‐
Smoking throughout (22)	6 (0.223)	‐	2 (0.341)	‐	6 (**0.021**)	4 (0.183)	6 (**0.011**)	5 (**0.043**)	6 (**0.015**)	5 (0.055)
Folic Acid^a^ (9)	1 (0.630)	‐	1 (0.398)	‐	1 (0.623)	‐	1 (0.572)	‐	1 (0.599)	‐
Coffee (15)	0 (−)	‐	0 (−)	‐	1 (0.803)	‐	0 (−)	‐	0 (−)	‐
Reported cold 6 months^a^ (75)	12 (0.089)	‐	6 (0.228)	‐	7 (0.660)	‐	10 (0.135)	‐	10 (0.185)	‐
Day nursery 8 months^a^ (11)	4 (**0.022**)	0 (−)	2 (0.119)	‐	3 (0.095)	‐	4 (**0.013**)	0 (−)	4 (**0.016**)	0 (−)

*Note*: All *p*‐values are for hypergeometric tests. *P*‐values in bold have <0.05 significance.

^a^ Where exposures are hypothesized to increase risk, the same direction of methylation change is expected; however, where exposures are hypothesized to be protective, an opposing direction of methylation change is expected.

Where constitutively across all subtypes there was significant overlap with DMCs associated with maternal radiation exposure during pregnancy (Table 1), subtype analysis also suggested a significant DMC overlap for most subtypes (7/10) (Table 3). However, after assessing the directionality of methylation change, only the *KMT2A‐r (MLL)* subtype was observed to have concordant methylation not likely to be due to chance (*p* = 0.044, Table 3). Similarly, for maternal alcohol‐associated methylation, where constitutive methylation across subtypes was observed to significantly overlap, for specific subtypes, this relationship was only observed for T‐ALL (*p* = 0.005), *ETV6::RUNX1* (*p* = 0.028) and undefined leukaemias (*p* = 0.040), with only T‐ALL (*p* = 0.008) and *ETV6::RUNX1* (*p* = 0.045) subtypes having significantly concordant directional methylation changes overlapping (Table 3). For reported colds, where significantly overlapping DMCs were observed with constitutive methylation across all subtypes, subtype‐specific analysis only revealed a statistically significant finding for *KMT2A‐r (MLL)* (Table 3, *p* = 0.042), where all DMCs were discordant for directionality of methylation change, which would be expected for an exposure considered protective.

Where previously DMCs associated with day care attendance were not observed to have any overlap with constitutional methylation across all subtypes (Table 1), significant overlaps were observed for T‐ALL (*p* = 0.048), *KMT2A‐r (MLL)* (*p* = 0.006), dic(9::20) (*p* = 0.037), *ETV6::RUNX1* (*p* = 0.022), undefined (*p* = 0.013) and non‐recurrent (*p* = 0.016) subtypes (Table 3). As we hypothesized day care attendance as a protective exposure, we assessed the opposing directionality of methylation between exposure and ALL but observed none, that is all methylation changes were in the same direction, which was not the expected directionality. Similarly, smoking throughout pregnancy was not observed to have any overlap with constitutional methylation across all subtypes, but for *KMT2A‐r (MLL)* (*p* = 0.004), dic(9:20) (*p* = 0.014), iAMP21 (*p* = 0.021), undefined (*p* = 0.011) and non‐recurrent (*p* = 0.015) subtypes, significant overlaps between smoking‐related methylation and disease‐associated methylation were observed (Table 3). When considering the directionality of methylation change, however, only *KMT2A‐r (MLL)* (*p* = 0.018) and undefined (*p* = 0.043) were unlikely to be due to chance. We also compared smoking‐associated DMCs reported in a meta‐analysis with subtype‐specific methylation (Table 4). When comparing constitutive methylation changes across all subtypes, there was no significant overlap (Table 2), but when analysed individually, significant overlaps were found for each of the 10 subtypes (Table 4). However, when considering the directionality of methylation change, there were no significantly overlapping concordant methylation changes related to maternal smoking and any of the individual subtypes (Table 4).

**TABLE 4:** Number of DMCs with altered methylation due to an ALL risk exposure reported in published 450 K studies (FDR corrected and cell type adjusted) and the overlapping number of DMCs also observed to have altered methylation in individual ALL subtypes.

ALL subtype (number of associated DMCs)	T‐ALL (58157)		*KMT2A‐r (MLL)* (31403)		*dic(9::20)* (53680)		HEH (42779)		*TCF3::PBX1* (21799)	
Exposure (number of associated DMCs)	Overlapping DMCs	Concordant DMCs (*p‐*value)	Overlapping DMCs	Concordant DMCs (*p‐*value)	Overlapping DMCs	Concordant DMCs (*p‐*value)	Overlapping DMCs	Concordant DMCs (*p‐*value)	Overlapping DMCs	Concordant DMCs (*p‐*value)
Sustained maternal smoking (6073)	929 (**5.03 × 10** ^**−6**^)	542 (1)	634 (**1.21 × 10** ^**−20**^)	287 (1)	943 (**6.41 × 10** ^**−14**^)	538 (1)	732 (**5.49 × 10** ^**−9**^)	391 (1)	476 (**1.49 × 10** ^**−21**^)	287 (0.846)
Maternal plasma folate^a^ (443)	123 (**9.43 × 10** ^**−16**^)	116 (**4.68 × 10** ^**−13**^)	57 (**1.78 × 10** ^**−5**^)	52 (**4.0 × 10** ^**−4**^)	92 (**3.58 × 10** ^**−7**^)	82 (**1.1 × 10** ^**−4**^)	55 (**0.043**)	41 (0.677)	39 (**5.3 × 10** ^**−4**^)	33 (**0.016**)

ALL subtype (number of associated DMCs)	*ETV6::RUNX1* (45589)		*BCR::ABL1* (23871)		iAMP21 (44726)		Undefined (39262)		Non‐recurrent (42109)	
Exposure (number of associated DMCs)	Overlapping DMCs	Concordant DMCs (*p‐*value)	Overlapping DMCs	Concordant DMCs (*p*‐value)	Overlapping DMCs	Concordant DMCs (*p‐*value)	Overlapping DMCs	Concordant DMCs (*p‐*value)	Overlapping DMCs	Concordant DMCs (*p‐*value)
Sustained maternal smoking (6073)	822 (**1.75 × 10** ^**−14**^)	465 (1)	506 (**2.21 × 10** ^**−20**^)	285 (0.997)	949 (**6.28 × 10** ^**−39**^)	549 (0.999)	611 (**0.002**)	336 (1)	682 (**2.41 × 10** ^**−5**^)	415 (1)
Maternal plasma folate^a^ (443)	71 (**2.0 × 10** ^**−4**^)	62 (**0.011**)	35 (**0.020**)	28 (0.243)	99 (**9.99 × 10** ^**−14**^)	88 (**1.34 × 10** ^**−9**^)	63 (**2.2 × 10** ^**−4**^)	56 (**0.007**)	71 (**1.75 × 10** ^**−5**^)	63 (**0.001**)

*Note*: All *p*‐values are for hypergeometric tests. *P*‐values in bold have <0.05 significance.

^a^ Where exposures are hypothesized to increase risk the same direction of methylation change is expected; however, where exposures are hypothesized to be protective, opposing directions of methylation change are expected.

Finally, we also analysed subtype‐specific methylation in relation to methylation associated with maternal plasma folate using data from a meta‐analysis. Where constitutively across all subtypes there was a significant overlap with DMCs also observed to be altered in response to maternal plasma folate during pregnancy (Table 2), subtype analysis corroborated these findings and suggested that for all subtypes there was a significant overlap between DMCs with altered methylation for each subtype and in response to maternal plasma folate (Table 4). When the directionality of methylation changes was considered, 8/10 subtypes were observed to have significantly overlapping discordant methylation patterns between maternal plasma folate and subtype‐specific associated methylation, which would be anticipated if maternal folate exposure is protective.

### 3.3 Comparison of KEGG pathway analysis across subtype‐specific‐associated methylation

Analysis revealed that between 65 and 90 KEGG pathways are likely to be significantly affected by differential methylation observed for specific subtypes (Tables S2 and S3), 49 of which were common across all subtypes. Pathways observed to be common between all subtypes included those related to signalling (i.e. MAPK, Ras, Wnt, Hippo) and cancer‐related pathways (i.e. pathways in cancer, proteoglycans in cancer, breast cancer). While most subtypes were observed to have methylation changes resulting in at least one or more KEGG pathways being unique to the subtype (Tables S2 and S3) *KMT2A‐r (MLL)*, dic(9::20) and non‐recurrent subtypes did not have any unique KEGG pathways associated with their differential methylation. While some unique pathways may plausibly relate to disease phenotype (i.e. TNF and HIF‐1 signalling in T‐ALL^31^ and ABC transporters in *BCR::ABL1* ^32^) others could potentially be artefacts.

### 3.4 Comparison of pathway analysis between subtype‐specific‐associated methylation and exposure‐associated methylation

Due to the limited number of methylation changes in response to some exposures, pathway analysis was only possible for maternal radiation during pregnancy, maternal plasma folate during pregnancy^26^ and maternal sustained smoking during pregnancy.^27^

One KEGG pathway was suggested to be affected by changes in methylation associated with maternal exposure to radiation during pregnancy (Tables S2 and S4a). Compared to pathways likely to be altered in response to methylation changes in ALL, this ‘MAPK signaling pathway’ was also observed across all subtypes (Tables S2 and 5).

**TABLE 5:** KEGG pathways plausibly altered in response to differential methylation associated with an exposure and also plausibly altered in response to differential methylation in all ALL subtypes.

KEGG ID	KEGG pathway	Number of genes^a^	*p*‐value
Maternal radiation exposure			
hsa04010	MAPK signalling pathway	8	0.033186
Maternal plasma folate levels			
hsa04024	cAMP signalling pathway	8	0.005796
hsa04080	Neuroactive ligand‐receptor interaction	9	0.024993
hsa04550	Signalling pathways regulating the pluripotency of stem cells	6	0.012975
hsa04713	Circadian entrainment	6	0.002551
hsa04724	Glutamatergic synapse	6	0.005108
hsa04727	GABAergic synapse	5	0.011161
hsa04728	Dopaminergic synapse	5	0.040371
hsa04934	Cushing syndrome	6	0.017833
hsa05032	Morphine addiction	5	0.012039
hsa05217	Basal cell carcinoma	4	0.023681
Maternal smoking (sustained) during pregnancy			
hsa01522	Endocrine resistance	27	0.001499
hsa04010	MAPK signalling pathway	62	0.002578
hsa04014	Ras signalling pathway	58	5.68E‐05
hsa04015	Rap1 signalling pathway	57	3.34E‐06
hsa04020	Calcium signalling pathway	49	0.013898
hsa04022	cGMP‐PKG signalling pathway	35	0.027137
hsa04024	cAMP signalling pathway	46	0.012378
hsa04072	Phospholipase D signalling pathway	39	2.86E‐04
hsa04151	PI3K‐Akt signalling pathway	81	3.11E‐05
hsa04360	Axon guidance	49	2.28E‐05
hsa04390	Hippo signalling pathway	34	0.018943
hsa04510	Focal adhesion	47	0.001112
hsa04550	Signalling pathways regulating pluripotency of stem cells	33	0.008403
hsa04713	Circadian entrainment	23	0.022371
hsa04725	Cholinergic synapse	28	0.006041
hsa04727	GABAergic synapse	22	0.016531
hsa04728	Dopaminergic synapse	29	0.025937
hsa04750	Inflammatory mediator regulation of TRP channels	22	0.044468
hsa04810	Regulation of actin cytoskeleton	49	0.002079
hsa04921	Oxytocin signalling pathway	38	0.001316
hsa04925	Aldosterone synthesis and secretion	24	0.013336
hsa04928	Parathyroid hormone synthesis, secretion and action	30	4.78E‐04
hsa05032	Morphine addiction	25	0.00244
hsa05165	Human papillomavirus infection	66	0.00724
hsa05200	Pathways in cancer	107	3.69E‐04
hsa05205	Proteoglycans in cancer	45	0.005129
hsa05226	Gastric cancer	32	0.02563

^a^ Refers to the number of genes on the pathway with altered methylation in response to the relevant exposure.

Fifteen KEGG pathways were observed to be significantly affected in response to methylation changes associated with maternal plasma folate levels during pregnancy (Tables S2 and S4b). Compared to pathways likely to be altered in response to methylation changes in ALL, 10/15 KEGG pathways were also observed across all 10 subtypes (Table 5), with 3 further KEGG pathways observed in at least 5 subtypes (Tables S4 and S4d).

Ninety KEGG pathways were observed to be significantly affected in response to methylation changes associated with sustained maternal smoking during pregnancy (Tables S2, S4, and S4c). Compared to pathways likely to be altered in response to methylation changes in ALL, 27/90 KEGG pathways were also observed across all 10 subtypes (Table 5), with 57/90 KEGG pathways highlighted as potentially associated with maternal smoking to also be potentially associated with at least one ALL subtype (Tables S4 and S4e).

## 4 DISCUSSION

Here, we performed a CpG‐level (DMC) analysis, corroborating our earlier findings suggesting that altered DNA methylation associated with maternal radiation exposure, alcohol intake, and plasma folate during pregnancy is also present in overt disease at a rate higher than expected by chance and may therefore contribute to disease aetiology. Meanwhile, where previously reported cold symptoms were found not to have significantly overlapping gene‐level methylation in common with ALL, the observed commonality between DMCs was significant. Of these specific DMCs, several could plausibly be involved in the causal pathway towards leukaemia. cg17470837 resides within the *JAKMIP1* gene (Tables S5, S5a, and S5b), which may activate Wnt signalling^33^ and has been suggested to be involved in JAK signalling, both of which are perturbed in leukaemia.^34^ cg04016431 is located in the *TRIM31* gene body, which has been implicated in cancers, including leukaemia.^35^ cg19929126, located within the *TRIL* transcription start site, encodes a protein with a functional role in the Toll‐like receptor 4 (TLR4) complex.^36^ Both TRIM31 and TLR4 are involved in the innate immune response and thus cytokine signalling that has previously been implicated in leukaemic progression.^35^, ^37^ Therefore, it is plausible that the discordant methylation changes associated with reported cold symptoms compared to those in ALL could mechanistically contribute to disease prevention, a novel finding from this study. Overall, our updated DMC analysis corroborated the major significant findings from our earlier study and has the advantage of increased sensitivity to pinpoint the exact genomic position in which methylation is altered. The benefit of mapping exact DMCs associated with both exposure and outcomes is more likely to uncover highly specified, relevant biomarkers for further investigation.

We also investigated these relationships for individual ALL subtypes to elucidate which exposures may contribute to the development/progression of specific subtypes. Supporting findings observed across all subtype‐associated methylation changes, methylation associated with maternal plasma folate during pregnancy significantly overlapped with methylation changes in 8/10 individual subtypes. Folate is a key player in one‐carbon metabolism, required for the production of the universal methyl donor, S‐adenosylmethionine.^38^ Insufficient folate available for methyl group donation influences the maintenance of methylation patterns. Indeed, folate consumption prior to conception and during pregnancy has been associated with variation in newborn methylation, including sites associated with acute myeloid leukaemia and ALL.^3^, ^39^, ^40^ This evidence suggests that maternal plasma folate status is likely to play a role in influencing DNA methylation patterns associated with most, if not all, ALL subtypes, rather than specific subtypes.

Meanwhile, methylation associated with maternal alcohol intake significantly overlapped with methylation observed in the T‐ALL and *ETV6::RUNX1* subtypes, suggesting that DNA methylation may be an intermediate mechanism for the influence of alcohol on these subtypes specifically. Alcohol has been associated with an increased risk of specific T‐cell lymphomas,^41^ which may align with findings here for T‐ALL, but no known associations have been observed between alcohol and the *ETV6::RUNX1* subtype. Methylation associated with reported colds at 6 months also significantly overlapped with methylation in the *KMT2A‐r (MLL)* subtype. To our knowledge, there is currently no evidence associating infection history with this specific subtype; therefore, this is a novel finding.

The *KMT2A‐r (MLL)* subtype also had significantly concordant overlapping methylation with maternal radiation exposure during pregnancy. As it is recommended to limit radiation exposure during pregnancy, it could therefore be assumed that few mothers whose children have leukaemia were exposed to radiation. However, as published in our previous paper, in a healthy population 3.4% of pregnancies in the cohort were exposed to medical radiation; therefore, it is plausible that children with *KMT2A‐r* leukaemia could have been exposed to radiation *in utero*.^3^, ^42^ One study of therapy‐related ALLs, where leukaemias developed following cytotoxic or radiation treatment for a different malignancy, found 50% of those who had 11q23 abnormalities,^43^ which may support this finding. A further study, however, found that therapy‐related leukaemias following treatment with radiation therapy alone had higher rates of *BCR::ABL1* mutations compared to *MLL*.^44^ This may suggest that radiation may influence both these genetic lesions, but that radiation‐associated methylation changes may be particularly important in influencing the progression of the *KMT2A‐r (MLL)* subtype, perhaps via synergistic interaction with the initiating genetic lesion. This warrants further investigation.

Furthermore, methylation changes associated with *KMT2A‐r (MLL)* and undefined subtypes had significant overlaps with methylation changes associated with smoking throughout pregnancy reported in Timms et al. However, using data from a larger meta‐analysis of sustained smoking during pregnancy,^27^ no association was observed with any subtype, indicating this could be a chance finding. The larger data set used to determine the DMCs associated with maternal smoking during pregnancy could be considered more robust due to the number of mother–child pairs analyzed, that is 6685 versus 861. However, a high proportion of the DMCs found in significantly overlapping concordant DMCs identified between the *MLL* subtype and maternal smoking using the Timms et al. data were also observed in the meta‐analysis. Moreover, these DMCs all map to one gene with potential functional interest, that is *CYP1A1*, a cytochrome P450 protein whose expression is induced by polycyclic aromatic hydrocarbons in cigarette smoke.^45^ Whilst there is currently no epidemiological evidence that maternal smoking is associated with increased risk of the *KMT2A‐r (MLL)* subtype, foetuses from smoking mothers have been observed to have increased frequency of *11q23* rearrangements compared to non‐smoking mothers.^46^ Therefore, it could be plausible that smoking‐associated altered methylation may contribute to a range of molecular events that drive this specific subtype of childhood ALL.

The lack of significant overlapping DMCs for every subtype and risk exposure may suggest that DNA methylation is not the predominating intermediate mechanism by which every risk exposure influences leukemogenesis overall and, moreover, that specific risk exposures contribute differentially to risk for each subtype, dependent on their influence on the epigenome. Indeed, supporting this, where significant overlapping concordant methylation was observed between specific subtypes and risk factors, a high proportion of DMCs (between 48% and 85%) were *not* those DMCs constitutive across all ALL subtypes (Tables S5 and S5c). This emphasizes the importance of considering subtype‐specific methylation. We would suggest this analysis is hypothesis‐generating and useful to indicate which risk factors may be aetiologically specific for certain subtypes, and these associations warrant further investigation.

**FIGURE 1:**
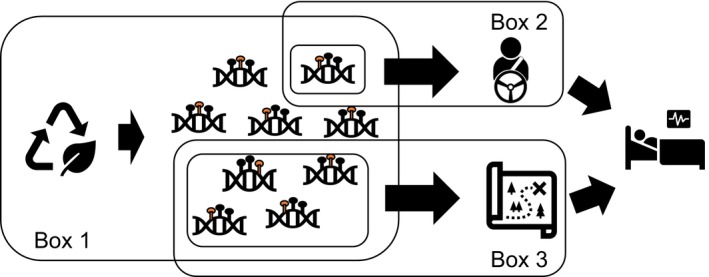
Schematic representation of the potential influence of environmentally associated DNA methylation on biological pathways involved in disease causality. Environmental exposures lead to a range of methylation changes, depending on the exposure (Box 1). Some of these environmentally orchestrated methylation changes may be ‘driver’ (Box 2) or ‘navigator’ (Box 3) methylation, or both, contributing to the causal disease pathway. Whilst ‘driver’ methylation changes would be in key DMCs/genes which are also present in disease and aligned directionally, navigator changes may be spread across a number of genes and not necessarily be in the same genes affected in the disease state, but instead influence biological pathways involved in disease causality.

Whilst each leukaemic subtype was unique in its methylomic profile, pathway analysis revealed that the potential overall impact of those changes could plausibly have similar functional effects. Forty‐nine KEGG pathways were found in common between all subtypes, with only a maximum of 10% of KEGG pathways unique to a subtype (Table S2). Comparing the influence of environmentally orchestrated methylation change on biological pathways with those observed in leukaemia, some key processes are likely to be affected, which could explain the potential routes through which these exposures influence leukaemia risk. Methylation associated with maternal radiation could plausibly influence the MAPK signalling pathway (Table 5), implicated in the pathogenesis of leukaemia.^47^, ^48^, ^49^, ^50^, ^51^ Methylation associated with maternal folate status may influence cAMP signalling, which is associated with the pathogenesis of BCP‐ALL,^52^ and signalling pathways regulating the pluripotency of stem cells. Whilst individually, methylation changes associated with maternal smoking do not significantly overlap with methylation change in leukaemia, there are several signalling pathways (MAPK, cAMP, Ras, Hippo, pluripotency of stem cells, etc.) that could be altered by smoking‐associated methylation change and are also likely to be influenced by the methylation patterns associated with leukaemia (Table 5). We therefore propose two plausible ways in which environmentally orchestrated methylation may be involved in the causal pathway towards ALL development. In the field of cancer epigenomics, the concept of ‘driver methylation’, whereby methylation of specific genes may be functionally important for cancer development, has been described.^53^ Conversely, ‘passenger methylation’ exists that is methylation change as a consequence of/in association with carcinogenesis, rather than contributing to the carcinogenic process. We suggest that environmentally associated methylation is likely to include both driver and passenger methylation, but *additionally* we propose ‘navigator methylation’ could contribute to disease development (Figure 1). In contrast to driver methylation, environmentally associated ‘navigator’ methylation may not occur in the same genes or DMCs as are observed in cancer itself, but instead, are a series of methylation changes that affect biological pathways implicated in disease development. Within the framework of this hypothesis, we therefore suggest that maternal radiation and folate intake during pregnancy may influence *both* driver and navigator methylation, whilst maternal smoking might only contribute to navigator methylation, which may reflect the conflicting epidemiological evidence regarding smoking as a risk factor for ALL. Moreover, we suggest navigator methylation is likely to influence specific signalling pathways, dependent on exposure. To evaluate the role of navigator methylation, further analysis utilizing mediation modelling could be performed to understand if DNA methylation influences the causal pathways between environmental exposure and childhood ALL.^54^ This analysis would require a data set that contains methylation data, exposure data and leukaemia outcomes within the same cohort, which is currently beyond the scope of this study. Due to the rarity of leukaemia, it would be difficult to generate large data sets that contain the required information; however, success has been observed through the integration of data from international cohorts, utilized in a recent study tracking epigenetic changes across the development span of childhood ALL.^55^ Such pooling of data sets may plausibly pave the way for future mediation studies.

This study has been beneficial in corroborating previous findings, generating new hypotheses concerning the aetiology of specific subtypes, and providing potential biological pathways through which environmentally orchestrated methylation may influence leukemogenesis, but there are some limitations. Firstly, not all known ALL subtypes were assessed here and would therefore warrant assessment in future studies. Despite using data collected from large‐scale prospective studies determining associations between risk exposures and DNA methylation, the robustness of the associations could be queried. Exposure measurements may be impacted by reporting bias as most were assessed through self‐reported questionnaires.^3^, ^27^, ^56^, ^57^ Misreporting due to the perceived stigma surrounding smoking, alcohol, and caffeine intake during pregnancy could lead to inaccuracies within exposure assessments.^58^, ^59^, ^60^ Many exposures had a small number of associated DNA methylation changes due to a lack of power, reducing the capacity to understand the influence of some exposures on methylation. Whilst rigorous statistical analysis aims to prevent false positive associations, often the quality of the exposure assessment is not optimal and based on estimations or proxy measurements. The use of biomarkers as exposure measurements (i.e. Joubert et al^26^ used maternal plasma folate as the measure of exposure) is likely to be more robust, but often data are not available and obtaining such measures is costly and implausible. Therefore, the development of more robust exposure‐methylation signatures would benefit future studies. Furthermore, it is likely that exposures do not influence methylation signatures independently, and future studies investigating the interaction between ‘protective’ versus ‘risk’ exposures would allow for better understanding of how real‐life exposures influence leukaemia development.

Here we provide evidence to suggest that exposure‐related methylation has the potential to contribute to the aetiology of ALL in a subtype‐specific manner via driver and/or navigator changes. Increased knowledge of the mechanisms and pathways through which the environment contributes to ALL risk strengthens the evidence for these exposures as risk factors. Such evidence provides public health policymakers and practitioners with further rationale for improving adherence to recommendations (i.e. taking folic acid supplements) and the development of new preventative policies (i.e. reducing medical radiation during pregnancy). Furthermore, understanding and knowledge of methylation events involved in leukemogenesis may lead to the development of early biomarkers, which may be developed for screening or monitoring to aid early diagnosis and improve patient outcomes.

## AUTHOR CONTRIBUTIONS

**Jessica R. Saville:** Data curation; writing – original draft; methodology; writing – review and editing. **Lisa J. Russell:** Supervision; writing – review and editing. **Kay Padget:** Supervision; writing – review and editing. **Akram Ghantous:** Writing – review and editing. **Jessica Nordlund:** Resources. **Jill A. McKay:** Conceptualization; writing – original draft; writing – review and editing; supervision; methodology.

## CONFLICT OF INTEREST STATEMENT

The authors declare no competing financial interests.

## ETHICS STATEMENT

This work has been approved by the IARC Ethics Committee (IEC 23‐36) and the Northumbria University Ethics Committee (49118).

## Supporting information


**Figure S1.** Schematic representation of the analysis framework.
**Table S2.** Overview of the number of KEGG pathways plausibly altered in response to differential methylation associated with an exposure or ALL subtype and the number of processes and pathways observed in both response to exposure and in an ALL subtype.

**Table S1.** List of environment CpGs and genes.

**Table S3.** KEGG pathway data for individual subtypes and comparison across subtypes.

**Table S4.** KEGG data for exposures and comparison with ALL subtypes.

**Table S5.** List of DMCs.

## Data Availability

Data sets utilized are in the public domain and included in the supplementary material. Further information is available from the corresponding author.
