# Supplementary material for: The potential role of environmentally associated DNA methylation in childhood acute lymphoblastic leukaemia subtypes
Source: Int J Cancer. 2025 Jun 9;157(8):1600–12. doi: 10.1002/ijc.35506 (PMC12375842; doi:10.1002/ijc.35506)
Supplement: Supplementary file 1 — Figure S1. Schematic representation of the analysis framework. Table S2. Overview of the number of KEGG pathways plausibly altered in response to differential methylation associated with an exposure or ALL subtype and the number of processes and pathways observed in both response to exposure and in an ALL subtype. [file IJC-157-1600-s001.pdf]

The potential role of environmentally-associated DNA methylation in childhood acute lymphoblastic leukaemia subtypes

Jessica R. Saville, Lisa J. Russell, Kay Padget, Akram Ghantous, Jessica Nordlund, Jill A. McKay.

Table of Contents:

Supplementary Table S1: List of environment CpGs and Genes. *Available as separate excel file.*

Radiation
Alcohol
Sugary caffeinated drinks
Smoking all pregnancy
Folic acid supplements
Coffee
Reported colds
Day nursery
Sustained smoking
Plasma folate

Supplementary Table S2: Overview of the number of KEGG pathways plausibly altered in response to differential methylation associated with an exposure or ALL subtype and number of process and pathways observed in both response to exposure and in an ALL subtype. *Page 3.*

Supplementary Table S3: KEGG Pathway Data for Individual Subtypes & comparison across subtypes. *Available as separate excel file.*

Table S3a. KEGG pathways which may be affected through altered DNA methylation in T-ALL.
Table S3b. KEGG pathways which may be affected through altered DNA methylation in KMT2A-r (MLL) ALL.
Table S3c. KEGG pathways which may be affected through altered DNA methylation in dic(9::20) ALL.
Table S3d. KEGG pathways which may be affected through altered DNA methylation in HEH ALL.
Table S3e. KEGG pathways which may be affected through altered DNA methylation in TCF3::PBX1 ALL.
Table S3f. KEGG pathways which may be affected through altered DNA methylation in ETV6::RUNX1 ALL.
Table S3g. KEGG pathways which may be affected through altered DNA methylation in BCR::ABL1 ALL.
Table S3h. KEGG pathways which may be affected through altered DNA methylation in iAMP21 ALL.
Table S3i. KEGG pathways which may be affected through altered DNA methylation in undefined ALL.
Table S3j. KEGG pathways which may be affected through altered DNA methylation in non-recurrent ALL.
Table S3k. Comparison of KEGG pathways altered in response to differential DNA methylation observed in different subtypes of childhood acute lymphoblastic leukaemia.

Supplementary Table S4: KEGG data for exposures and comparison with ALL subtypes. *Available as separate excel file.*

Table S4a. KEGG pathways which may be affected through altered DNA methylation in offspring in response to maternal radiation exposure during pregnancy.
Table S4b. KEGG pathways which may be affected through altered DNA methylation in offspring in response to maternal folate exposure during pregnancy.
Table S4c. KEGG pathways which may be affected through altered DNA methylation in offspring in response to sustained maternal smoking during pregnancy.
Table S4d. Comparison of KEGG pathways altered in response to differential DNA methylation observed in relation to maternal plasma folate during pregnancy and across different subtypes of childhood acute lymphoblastic leukaemia.

Table S4e. Comparison of KEGG pathways altered in response to differential DNA methylation observed in relation to sustained maternal smoking during pregnancy and across different subtypes of childhood acute lymphoblastic leukaemia.

Supplementary Table S5: List of DMCs. *Available as separate excel file.*

Table S5a. List of DMCs for increased risk exposures in which significant associations were observed via hypergeometric testing after accounting for directionality of methylation change
Table S5b. List of DMCs for protective exposures in which significant associations were observed via hypergeometric testing after accounting for directionality of methylation change
Table S5c Number of overlapping DMCs for those exposures and subtypes observed to have significant observations, including the number of DMCs also observed to be constituently methylated across all subtypes.

Supplementary Figure S1: Schematic representation of analysis framework. *Page 4.*

Supplementary Table S2. Overview of the number of KEGG pathways plausibly altered in response to differential methylation associated with an exposure or ALL subtype and number of process and pathways observed in both response to exposure and in an ALL subtype.

		T-ALL	*KMT2A-r (MLL)*	*dic(9::20)*	HeH	*TCF3::PBX1*	*ETV6::RUNX1*	*BCR::ABL1*	iAMP21	undefined	non-recurrent
Exposures	No. KEGG pathways (no. unique to subtype, % subtype-specific)	90 (9, 10%)	65 (0, 0%)	78 (0, 0%)	83 (5, 6%)	79 (2, 3%)	90 (1, 1%)	83 (1, 1%)	83 (2, 2%)	81 (1, 1%)	80 (0, 0%)
	No. KEGG pathways										
Maternal smoking (sustained)	90	47	37	38	41	40	50	48	46	39	43
Maternal plasma folate	15	12	11	12	12	13	13	13	11	12	12
Maternal radiation	1	1	1	1	1	1	1	1	1	1	1

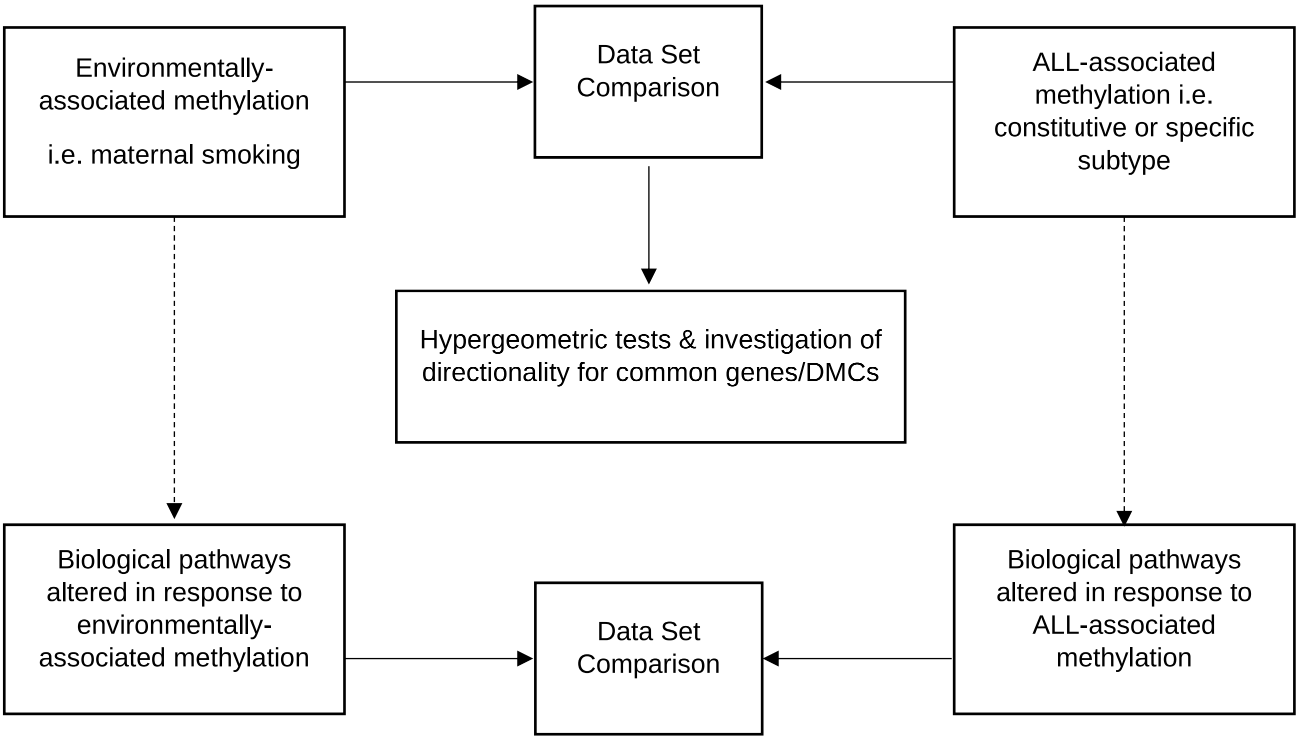

Supplementary Figure S1. Schematic representation of analysis framework. Environmental-associated methylation changes were compared with ALL-associated methylation changes at either gene or DMC level for a given exposure (e.g. maternal smoking), ALL subtype or constitutive methylation across all subtypes. Hypergeometric tests assess the probability of common genes/DMCs between data sets, taking the directionality of methylation into account. Individually, environmentally-associated methylation and ALL-associate methylation under-go pathway analysis (represented by the dotted line in the schematic) to determine biological pathways which may be altered in response to variable methylation in the given data set. Data sets are compared to discover if environmentally-associated methylation alters disease-related pathways.
